# Laypersons' Esthetic Perception of Different Dentogingival Characteristics Based on Smile Dynamics: Cross-Sectional Study

**DOI:** 10.1155/2024/5561640

**Published:** 2024-01-16

**Authors:** Adriana S. Malheiros, Julianne R. Barboza, Sebastião M. Pinheiro Neto, Daniela B. Dibai, Etevaldo M. Maia Filho, Célia Maio Pinzan-Vercelino, Meire C. Ferreira, Rudys R. J. Tavarez

**Affiliations:** ^1^Postgraduate in Dentistry, Ceuma University of Maranhão, São Luís, MA, Brazil; ^2^University center Ingá, Maringa, PR, Brazil

## Abstract

This study aimed to evaluate laypersons' esthetic perception of different dentogingival characteristics based on smile dynamics. Six voluntary models were selected, with only one presenting dentogingival characteristics within esthetic standards: white teeth, good alignment, and adequate gingival contour. Two videos were then produced. One video focused on the mouth, whereas the other focused on the entire face of the model, to ensure that the dynamics of the smile could be evaluated. For the evaluation, 200 laypeople were asked to rank the models from first to sixth place in their order of preference. Laypeople were required to justify their reasons for choosing the first and last places. The obtained data were subjected to descriptive and inferential statistical analyses. The results showed that smile and face dynamics influenced the perception of dentogingival characteristics and facial esthetics. No significant changes were observed. However, a gummy smile accompanied by dental alterations was observed in the video of the mouth and was masked by the facial set exposed in the video of the face. A diastema between the central incisors was decisive for negative evaluation of both the mouth and face. The standard model was rated as the most pleasing. Smiles and facial dynamics influence the perception of dentogingival characteristics. Smile and facial dynamics influence the perception of dentogingival alterations among Brazilian laypeople. Diastema is an alteration that interferes with the isolated evaluation of the lower third of the face and the facial set.

## 1. Introduction

Smile esthetics and good appearance have become increasingly important, with dental composition being a key factor [1]. Attractive people are considered intelligent, competent, and pleasant, making it easier for them to incorporate themselves into their social environments [2]. Currently, people are concerned about the appearance and attractiveness of their teeth and are exploring options for further cosmetic treatment. Concern about the esthetics of smiles has increased the demand for the field of cosmetic dentistry because of exposure to social media. As esthetic facial and dental procedures become popular, people demand ever-improving smile esthetics. In contrast, those with a less attractive appearance are more likely to suffer from low self-esteem and have greater difficulty relating to others [3].

The face is a part of the body that is frequently exposed; therefore, it is most frequently observed and evaluated by other people. Within the facial set, smiles become differential because they outline feelings that can add value to an individual through physical means [4, 5]. Despite being subjective, esthetic perceptions are generally influenced by sociocultural standards, emotional factors, and lived experiences [6]. Understanding esthetic perception is extremely important for dentistry professionals, as it can be used as a reference to establish diagnosis and prognosis and guide treatments.

Smile esthetics promote different facial expressions, and gingival tissue is of great importance for this evaluation [7]. Dentogingival alterations frequently occur in natural dentition, making them important in this context. Several factors can interfere with the satisfactory esthetics of a smile that is considered nongingival. Among these nongingival aspects are altered passive dental eruption, reduced clinical dental crown, exaggerated growth of the maxilla in the vertical direction, and decreased thickness of the upper lip [8].

Both experts and patients reported that smile esthetics are impaired when there is excessive gingival exposure during smiling, which is associated with the position of the upper lip in relation to the gingival margin and influenced by the sex and age of the individual [9]. The dental anatomical position in relation to the gingiva, lips, and facial pattern of each patient suggests the shape and contour of the teeth, which are specific for better esthetics of the smile line [10]. Older people have a lower smile line with greater exposure of the lower teeth, compared to the young people, with aging there is a predominance of triangular teeth. In females, there is a predominance of straight lips and in males, there is a downward curvature of the lips [11]. Another aspect analyzed is the way to evaluate the smile, with smile dynamics being considered more realistic when the smile is evaluated [12].

Some studies [1, 13, 14] have sought to understand the importance of these alterations in the esthetic perception of both laypeople and dentists; however, most of these previous evaluations were conducted statically using photographs. Therefore, it is important to conduct studies to evaluate the esthetic perception of different smiles during movement. Based on these considerations, this study aimed to evaluate the esthetic perceptions of different dentogingival characteristics based on the smile dynamics. The null hypothesis was that smiles and facial dynamics did not influence the perception of dentogingival characteristics.

## 2. Materials and Methods

### 2.1. Study Design

This was a cross-sectional observational study that followed the STROBE guidelines. Data were collected in full accordance with the World Medical Association Declaration of Helsinki and approved by the Committee for the Protection of Human Participants of the local university (protocol number: 832,059).

For the selection of the model volunteers and video production, six academics aged between 19 and 25 years, mixed-race were selected as the “model volunteers”. All had different dentogingival characteristics, one of which was within esthetic standards: white teeth, good alignment, and adequate gingival contour [10].

Standardized videos were produced using an iPad (Apple Inc., Cupertino, CA., 2017 model, 16 GB) fixed to a tripod at 60 cm. The model volunteers were instructed to sing the same song in a relaxed manner and smile during the 30-s recording. The videos were produced in the frontal position with a white background. The video was edited using the iMovie program (Apple Inc., version 10.3) so that the voices of the models were not audible. All the shooting procedures were performed on the same day. Two videos were recorded for each participant. One video showed the lower third of the face with the teeth and lips exposed to the base of the nose (video of the lower third of the face), whereas the other video showed the entire face (face video).

For the selection of the evaluators and evaluation of the videos, a sample calculation was carried out considering the six voluntary models, alpha value equal to 0.05, and the power of the ANOVA test, for repeated measures for a factor of 0.95 to find the effect size of the independent variable on the dependent variable of 0.25 (effect average or typical). The result was a total of 192 evaluators, which was rounded to 200 (G Power, Heinrich-Heine Universitat, Dusseldorf, Germany).

The evaluators (200 Brazilian people with little or no knowledge in dentistry called laypeople) were randomly selected; they were university students, did not know the volunteers, were not from the field of dentistry, and had signed an informed consent form. The randomization method used was in a block of four and was performed using the sealed envelope TM (https://www.sealedenvelope.com/simple-randomiser/v1/lists).

The videos were presented for evaluation purposes. Each model was identified using the letters A–F (Table 1). The videos were organized according to dentogingival characteristics. Initially, the evaluators watched a video showing only the lower third of their faces. In ascending order, the evaluators indicated the model of their preference, attributing a score of one to the model that pleased them the most esthetically, and a score of six to the one that pleased them the least. Afterward, they were asked to justify the reason for choosing the first and last place. The researcher recorded each participant's choices and justifications. Next, videos of the faces were displayed. The selection process followed the same methodology used in the previous step. To avoid bias, the order and letters of the models differed between videos.

### 2.2. Statistical Analysis

The data were tabulated and subjected to descriptive and inferential analyses using the Friedman test and two-by-two comparisons using the Wilcoxon test with Bonferroni correction. This test was used to assess whether there was a significant difference between the scores attributed to the lower third of the face and the face assessments in the different evaluated models. The effect size was calculated using Kendall's W.

Kappa values were used to determine whether there was agreement between the judgments for the lower third of the face and the face and whether these judgments differed depending on the sex of the rater. In addition, the agreement between the mouth and face ratings for each model was evaluated.

The significance level adopted was 5%. SPSS (version 23.0; IBM, Armonk, NY, USA) was used for the statistical analysis.

## 3. Results

The average ages of the female and male evaluators were 21.77 (6.65) and 22.6 (5.53) years, respectively. The mean (standard deviation) of the scores attributed to the evaluations of the mouth and face videos was obtained (Table 2). The lowest averages were for models that were most pleasing to the evaluators, whereas the highest averages were for models that were least pleasing to the evaluators.

For the adopted significance level of 5%, there was no statistically significant difference among models C, D, and E for the video showing the lower third of the face. Regarding the justifications provided by the evaluators, the three groups tended to be equal in terms of white teeth, aligned teeth, and good gingival contour.

**Figure 1:**
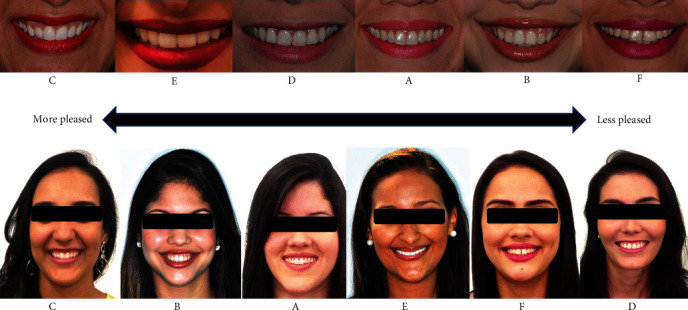
Scoring order in relation to the model volunteers you liked the most and the one you least liked in the video of the mouth and face.

For the face video, there was no statistical difference between models D and F, and models E and F, which had the highest scores. There was also no statistical difference between models A and C, and models B and C. Model C had the lowest average score and was deemed the most pleasing for both videos (Figure 1).

The effect size of the lower third of the face and face characteristics on the models' degree of attractiveness were analyzed using Kendall's W, and the values found were 0.372 for the mouth and 0.409 for the face, both considered moderate effects [15].

The weighted kappa showed low-agreement values (slight agreement) when comparing the evaluators' agreement when analyzing the mouth and face. Similarly, the sex of the evaluator did not influence the agreement of the assessment, as both obtained low and similar kappa values (slight agreement; Table 3).

Except for model F, which had a kappa value of 0.253 (fair agreement), the results were discordant between the mouth and face, regardless of the model evaluated and the evaluator's sex. This result showed that there was no relationship between what participants scored on the mouth assessment and what they scored on the face (values ranged between 0.008 and 0.090, with slight agreement; (Table 3).

## 4. Discussion

The null hypothesis was that smile and face dynamics did not influence the perception of dentogingival alterations. This was rejected because alterations, such as diastema and high smile exposure, were found to displease the evaluators. This interference was reduced when the dynamics of smiles with movement and facial exposure were evaluated. Many studies [1–4, 8, 11, 13, 16–18] have statistically evaluated smiles. However, some studies that have evaluated the dynamics of the smiling state have found that the beauty of a smile emanates from its movement and from the dynamic integration of the teeth, gums, lips, and face. As such, static photos are inadequate for evaluating the perception of one's smile, and the perception of esthetics in motion differs from that in the static view [12, 19, 20].

Dynamism creates an esthetic perception that differs from that observed in a static set. Among the reasons reported by the evaluators for choosing the standard model (C) as the one that was most pleasing were characteristics such as muscle movement and the way the face moved when speaking, smiling, and conveying facial expressions [4].

By analyzing the individuality of the models, we found that model B, which had a gingival exposure of approximately 3–4 mm, had lower scores in the video of the exposure of the lower third of the face, where the evaluators stated that the large exposure of the gingiva was displeasing. However, when the video of this model's face was evaluated, most respondents did not focus on the gummy smile but on the set, which may have been the reason for the lower score [16]. Some studies have shown that evaluating gingival exposure using videos and smile dynamics can provide more repeatable smiles and more reliable data, which may explain this result [12].

Another important aspect is that the accentuated gingival display was clearly perceived by the laypeople, both in the video of the mouth and in the dynamism of the face. Studies by Al Takiet et al. [13] and Ousehal et al. [17] have shown that there is a loss of attractiveness when the distance between the gingival margin and lip is greater than 4 mm. In contrast, Mokhtar et al. [18] used the image of a smile with an exposure of 5 mm, and only 41% of laypeople rated it as unattractive. Al Taki et al. [13] found that laypeople find an exposure of 1–2 mm of the gingiva more attractive.

Model C was unanimously evaluated as the most pleasing for both videos. The evaluators said she had “beautiful, white, well positioned and aligned teeth” and “marking eyes and face.” The attractiveness of different smiles has been evaluated previously. Smiles with a high or medium anterior smile line, parallel smile arc, upward curvature of the upper lip, second premolars as the most exposed posterior teeth, and dynamic symmetry were considered the most attractive [13].

The volunteer in model D, who had misaligned lateral incisors and premolars and altered gingival zenith, lay people in the video of the face rather than in the video of the lower third of the face. This is because changes in the dental position and gingival zenith are noticeable in the dynamism of the face, especially in the case of the anterior teeth. Studies by Nomura et al. [8] and Sobral et al. [21] have shown that asymmetric gingival zeniths or differences in tooth height are less attractive. Gingival zenith differences or differences in tooth height greater than 1 mm were noticeable for smile attractiveness.

The model A volunteer, who had a change in height between the central incisors, the evaluators rated the video of the lower third of the face as less pleasant than the video of the face. The evaluators stated that although there was a difference in the height of the central incisors in the video of the face, this difference was less noticeable. Another study by Wang et al. [22] found that laypeople could reliably identify attractive and unattractive smiles when viewing the lower third of a face.

The model E volunteer, who has a slight midline deviation, was among the best evaluated in the first video since she has “white and aligned teeth,” and the deviation was not mentioned by any of the interviewees. In the video of the face in which the dynamism of the set was evaluated, other facial aspects were considered to have increased the scores. It was previously reported that a line deviation of up to 3 mm is not considered unsightly by laypeople, which is consistent with the current study [21].

Model F obtained a high mean score for both videos, with the evaluators highlighting the diastema, showing that certain alterations did not go unnoticed by the group. The diastema in the central incisor region has a great impact, as has already been shown in the previous studies [3, 13, 23].

Another important point was that, when showing the faces of the volunteer models, the evaluators attributed justifications that went beyond physical appearance, such as personality traits and feelings, which further reinforces the importance of using videos and dynamic media in esthetic evaluations of any type, as the movement brings about a new perception of what is observed [4].

Regarding the sex effect of lay evaluators, the findings of this study showed that there was no significant difference in esthetic judgments between female and male evaluators, which means that dentofacial alterations were observed in the same way in both sexes when dynamically evaluated, which is consistent with the results of other studies [16]. However, other studies have found that females are more critical when evaluating aspects related to smiles [2].

There is extensive literature on smile esthetics. Nevertheless, many previous studies used induced and static smiles, which could introduce bias. These studies have disregarded the time dimension as a significant parameter. Most captured (or attempted to capture) the peak or maximum extent of a smile using a single photograph. Thus, in addition to the uncertainty in acquiring an image at the correct time, this record contains no information regarding the evolution of movements over time [24].

Among the limitations of the study is the fact that it was carried out with mixed-race and young Brazilian people. We suggest other studies that evaluate this topic with people of different ages and genders. In addition to other racial types, as these factors can influence the esthetics of the smile.

## 5. Conclusion

Smile and face dynamics influence the perception of dentogingival alterations in Brazilian laypeople. These alterations were not observed when evaluated dynamically within the harmonic facial set. Diastema was found to interfere with the isolated evaluation of the lower third of the face and facial set.

## Figures and Tables

**Table 1:** Dentogingival characteristics of the models.

Model	Dental–gingival characteristics
A	Difference in cervical height of upper central incisors (1 mm)
B	Gummy smile with 3–4 mm exposure
C	Standard (well-balanced face, between eyes, nose, and mouth. Clear, well-aligned teeth and gingival contour on the upper lip line)
D	Lateral incisors and premolars with gingival zenith change and slightly misaligned
E	Midline deviation 2 mm to the left
F	Diastema between the central incisors of 2 mm

**Table 2:** Mean values (standard deviation) and median of the scores distributed according to the models and evaluations of the lower third of the face and face with the respective comparisons (Wilcoxon test with Bonferroni correction).

Model	Lower third of the face average (sd); median	Face average (SD); median
A	3.76 (1.30); 4^a^	2.90 (1.25); 3^a^
B	4.69 (1.28); 5^b^	2.49 (1.32); 3^ab^
C	2.41 (1.34); 2^c^	2.03 (1.19); 2^b^
D	2.66 (1.30); 3^c^	4.93 (1.23); 5^c^
E	2.51 (1.40); 2^c^	4.10 (1.51); 4^d^
F	4.96 (1.47); 6^b^	4.57 (1.36); 4^cd^

Different letters in the vertical direction indicate significant differences (*P* < 0.05).

**Table 3:** Weighted kappa results for comparing the judgments for mouth vs. face between female and male raters and depending on the model type.

Lower third of the face vs. face	0.063
Females	0.060
Males	0.066
Lower third of the face vs. face by model	
A	0.034
B	0.008
C	0.086
D	0.006
E	0.090
F	0.253

## Data Availability

The datasets used and/or analyzed during the current study are available upon reasonable request to correspondence author.
